# Successful use of therapeutic anticoagulation therapy in two patients with moderate stroke from the second day of onset: A case report and literature review

**DOI:** 10.1016/j.amsu.2022.104726

**Published:** 2022-09-16

**Authors:** Mostafa Meshref, Nour Shaheen, Rehab Adel Diab, Mariam Tarek Desouki, Yara Amro, Shiamaa M. Khairat, Mohamed Ali, Mahmoud Galal Ahmed

**Affiliations:** a Neurology Department, Faculty of Medicine, Al-Azhar University, Cairo, Egypt; b Faculty of Medicine, Alexandria University, Alexandria, Egypt; c Faculty of Medicine, Al-Azhar University, Cairo, Egypt; d Pharmacist Ministry of Health, Cairo, Egypt; e Division of Neurology Medicine, Department of Internal Medicine, King Khalid Hospital Hail, Saudi Arabia; f Neurology Department, Louisiana State University, Shreveport, LA, USA

**Keywords:** Stroke, Ischemic stroke, Anticoagulants, Atrial fibrillation, Warfarin

## Abstract

**Introduction and importance:**

Hemorrhagic transformation of ischemic stroke is one of the most traumatic consequences of ischemic stroke. Therefore, deciding the optimal time for anticoagulant application and its effect on clinical outcome, recurrence and risk for hemorrhagic transformation are still in quarry. The European Heart Rhythm Association recommends the usage of anticoagulants after 3–4 days after a mild stroke, 6 days after moderate stroke and 12 days after a severe stroke.

**Case presentation:**

In our case report, we present two patients who started full therapeutic anticoagulation of low molecular weight heparin from the first day after moderate ischemic stroke, warfarin was added later guided by INR and discharged on oral anticoagulants for associated AF. They improved clinically with improved motor function for both upper and lower limbs, sensation and gaze without any complication followed by serial CT.

**Clinical discussion and conclusion:**

As a result of this case report, clinical improvement has not been associated with hemorrhagic sequelae of anticoagulant administration on the first day. At this point, we recommend conducting a trial to study the effect of early application of anticoagulants from the first day on clinical outcome, recurrence, and hemorrhagic transfusion of stroke.

## Highlights

• The hemorrhagic transformation of ischemic stroke is one of the most traumatic outcomes.
• Therefore, determining the optimal time to apply anticoagulants.
• And their impact on clinical outcome, recurrence, and hemorrhagic transformation remains challenging.
• According to this case report, there are no hemorrhagic sequelae associated with the first day of anticoagulant therapy.
• Anticoagulation recommendations should therefore be supported or rejected based on more research.

## 1 Introduction

Atrial fibrillation (AF) carries a substantial risk of recurrence of ischemic stroke [1]. Patients with AF have a 5-folded risk of stroke compared to healthy individuals [2]. One of the most devastating consequences of ischemic stroke is hemorrhagic transformation (HT) which affects about 8.5% of the ischemic stroke population [2,3]. Anti-coagulant use in cardio-embolic ischemic stroke is a double-edged weapon as its early use can increase the risk of HT, and delayed use can increase the risk of ischemic stroke recurrence. Therefore, personalized time of anticoagulant introduction according to the status of each patient is one of the most preferred methods in the management of AF patients. Size of infarct, mass effect, hyperglycemia, anticoagulant therapy before admission, and haemoglobin concentration collected within the first 3 days of admission are considered independent predictors for HT [4].

Oral anticoagulants can be chosen based on the presence of valvular disease (valvular vs. non-valvular AF). Valvular AF refers to the prosthetic valve or rheumatic mitral valve stenosis [5]. The European Heart Rhythm Association (EHRA) released a consensus stating that the best choice for non-valvular AF is novel oral anticoagulants (NOACs), while the best for valvular AF is vitamin K antagonist (warfarin) [6,7]. Patients with valvular AF carry a higher thromboembolism risk and are more likely to form the thrombus outside the left atrium for unknown causes, in contrast to non-valvular AF which are at lower risk and mostly from the thrombus in the left atrium itself [5]. In this report, we presented we present two cases of ischemic stroke with AF in line with the SCARE 2020 criteria [8].

## 2 Case one

A 38-year-old male, without any history of chronic illness, not diabetic nor hypertensive, presented with acute onset, one-day duration left side weakness (upper limb more than the lower limb) The patient presented with a deviation of the mouth to the right, with partial gaze preference to the right side.

In addition, the patient was drowsy and suffering from left hemi hypoesthesia, including facial symptoms. His NIHSS score was 14 which is going with moderate stroke severity (level of conscious 1a arousable by minor stimulation = 1, 1b answers one question correctly = 1, performs neither task correctly = 2, partial gaze palsy = 1, partial hemianopia = 1, partial facial paralysis = 2, motor left arm weakness with no effort against gravity = 3, motor left leg weakness with drift = 1, no limb ataxia = 0, mild to moderate sensory loss = 1, no aphasia = 0, no dysarthria = 0 and finally visuospatial and personal in attention = 1). His modified Rankin scale (MRS) at admission was 4.

The patient had a history of a transient ischemic attack 2 weeks before onset in the form of weakness on the left side. The patient sought medical advice and was prescribed (81mg aspirin PO od), but no stroke workup was done due to insurance problems.

On general examination, the patient was also found to have AF and a murmur heard at the apex, which suggested tight mitral stenosis. Echocardiography was done at the emergency department which shows evidence of mitral stenosis (rheumatic heart disease) with left atrial enlargement and evidence of left atrial thrombus with a possibility of left atrial appendage for further surgical intervention later. The cardiologist recommended starting full anticoagulant therapy.

**Fig. 1:**
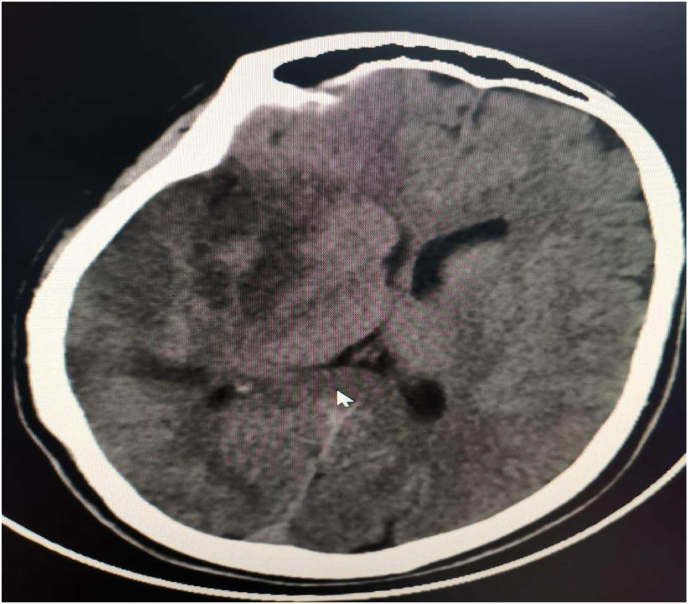
Non-contrast CT brain showed right tempro-parietal hypodense area denonting ischemic insult.

In addition to that, she had pallor and coldness of both lower limbs we consulted vascular surgery and bedside doppler done which were highly suggestive of lower limb ischemia and they recommended full anticoagulation therapy and CT angiography of both lower limbs confirmed the presence of bilateral lower limb ischemic with a sluggish flow with also evidence of right kidney infarction. Ct brain (Fig. 1) shows right parietal infarction.

The patient was admitted to the intermediate care unit, and he was vitally stable his blood pressure, pulse and random blood sugar were checked, and all results were normal.

Also, routine lab investigations (including complete blood count, kidney, and liver functions all within normal. In addition to that, the thrombophilia screen and lipid profile were normal.

The patient started anticoagulation therapy. The patient was placed on enoxaparin 6000 IU SC bid for 3 days followed by warfarin (started with 3 mg PO od) based on the INR and continued on this regimen for 3 days. The patient was discharged home after 10 days on warfarin 6 mg PO od and his INR was 2.6.

Clinically, the patient was able to walk without support, with the improvement of his gaze and sensation, and improved his upper limb motor power. His MRS was 1.

## 3 Case two

A 37-year-old female, married, without any chronic illness, not diabetic nor hypertensive, not on medications or taking oral contraceptives. She was presented with acute left side weakness and no intracerebral bleeding was found on the CT brain of the patient.

After 1 h of onset, RTPA was begun, and the patient was transferred to our facility.

At that time, the patient was drowsy with left side weakness and deviation of angle of mouth to the right side with hemi hypoesthesia of left side including the face without gaze affection.

Her NIHSS score was 11 which is going with moderate stroke severity (level of conscious 1a arousable by minor stimulation = 1, 1b answers one question correctly = 1, performs neither task correctly = 1, no gaze palsy = 0, partial hemianopia = 1, partial facial paralysis = 2, motor left arm weakness with no effort against gravity = 3, motor left leg weakness with drift = 1, no limb ataxia = 0, mild to moderate sensory loss = 1, no aphasia = 0, no dysarthria = 0 and finally visuospatial and personal in attention = 1). Her MRS at admission was 4.

The patient had a history of complaining of easy fatigability associated with dyspnea with mild usual activity over the past three years. However, there was no history of rheumatic fever or long-acting penicillin use.

She was admitted to ICU. Her blood pressure, pulse, and random blood sugar were all normal. In addition, routine lab tests (including complete blood count, kidney tests, and liver tests) were all within normal ranges. However, her ECG showed AF. Further investigations including the thrombophilia screen and lipid profile were both normal.

The echocardiography was done and revealed a picture of rheumatic heart disease with moderate mitral stenosis, trivial mitral regurgitation, mild to moderate aortic regurgitation, and dilated left atrium with an ejection fraction of 35%.

**Fig. 2:**
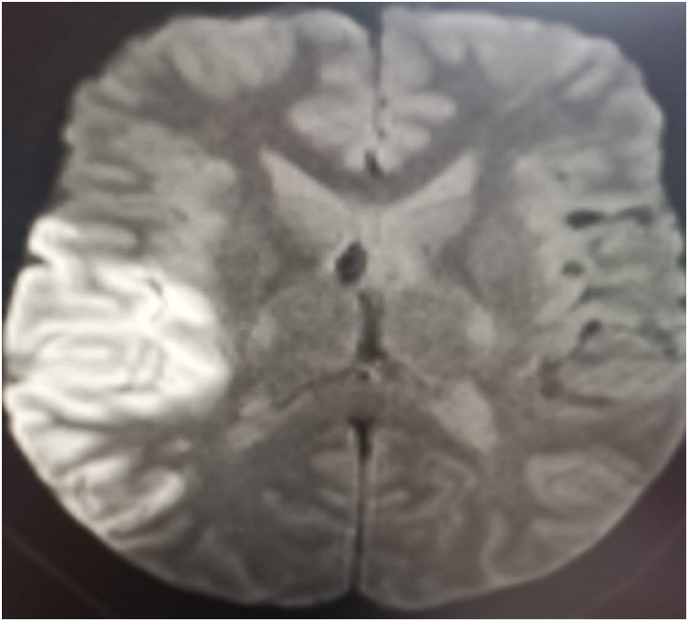
MRI brain with diffusion weighted images showed hyperintense lesion in the right parieto-occipital denoting acute infarction.

MRI brain was done in figure (2) which showed a right parieto-occipital hyperintense lesion denoting acute stroke.

The patient started anticoagulation therapy. We put the patient on enoxaparin 6000 IU SC bid and continue for 5 days and added warfarin (started with 3 mg PO od) guided by INR.

The patient was discharged home after 14 days on warfarin 4 mg PO od and his INR was 2.8.

**Fig. 3:**
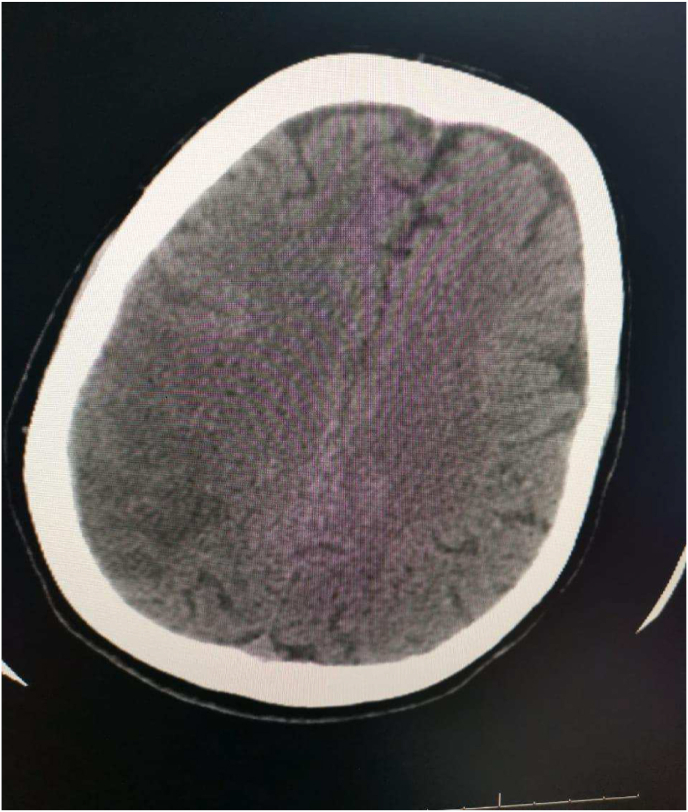
Ct brain follow-up showed regression of the right parieto-occipital hypo dense lesion.

During follow-up CT brain imaging (Fig. 3), the patient improved clinically and was discharged on oral anticoagulation. Her MRS became 1.

MRI brain (Fig. 2)

## 4 Discussion

Acute ischemic stroke (AIS) is a nonspecific state of brain injury with neuronal dysfunction, occurring because of the sudden loss of blood circulation to an area of the brain, typically in a vascular territory, leading to a corresponding loss of neurologic function. A thrombotic or embolic occlusion of a cerebral artery is usually the cause of acute ischemic stroke [9].

Patients with acute stroke and AF (AF) are reported to face a 0.1%–1.3% daily risk of early recurrence. Treatment with anticoagulants is the most effective way to prevent recurrent ischemic stroke, but the acute hemorrhagic transformation in patients with AF is a major concern, so timing the onset of anticoagulant therapy accordingly is essential [10]. Although AF patients rarely suffer transient ischemic attacks (TIAs), and little information is available on their prognosis and treatment response, the first case had TIA two weeks ago and showed a successful response [11].

There are variable national and international recommendations on the time we should start anticoagulants after ischemic stroke in AF patients [12]. American Heart Association/American Stroke Association (AHA/ASA) recommends starting anticoagulants within 4–14 days in most patients considering more delay in patients with HT [13]. European Society of Cardiology (ESC) and EHRA recommendations are to give anticoagulants in a timeline base on the degree of stroke severity that is defined by the National Institute of Health Stroke Scale (NIHSS) scale as the following: start 1 day after the onset of the transient ischemic attack, 3 days after minor stroke (NIHSS score <8), 6 days after mild stroke (NIHSS score 8–15), and 12 days after severe stroke (NIHSS score >15) [14,15].

According to the system of categorizing stroke developed in the multicenter Trial of ORG 10172 in Acute Stroke Treatment (TOAST), ischemic strokes are divided into the following 3 major subtypes: large artery, small vessel or lacunar, and cardio-embolic infarction.

Our area of interest in our presented case report is the ischemic stroke caused by cardio-embolic infarction, more specifically caused, by AF, which is a heart condition, characterized by an irregularly, irregular, rapid atrial rhythm.

It was reported that cardiogenic emboli are a common source of recurrent stroke, accounting for up to 20% of acute strokes and having the highest 1-month mortality [16].

As hypertension, diabetes mellitus, and high cholesterol, are considered risk factors in the development of atherosclerotic and cardiac disease, contributing to the development of acute ischemic stroke, adding to the coagulation factors abnormality, a throughout history has to be taken from the patient when presenting to the ER, to exclude the presence of any co-risk factors that may aggravate the patient's condition. This was followed with our 2 patients presented in the case report we made, and it was found that laboratory investigations including complete blood count, kidney, and liver functions, the thrombophilia screen and lipid profile were all within normal [17].

Adding to that an echocardiography was done on both patients, to confirm the source of cardio-embolic stroke, and the following was found: left atrial enlargement and evidence of left atrial thrombus with a possibility of left atrial appendage for further surgical intervention later, and for the second one: a picture of rheumatic heart disease with moderate mitral stenosis, trivial mitral regurgitation, mild to moderate aortic regurgitation, dilated left atrium with an ejection fraction of 35%.

A detailed neurological and physical examination was done on both patients to establish the baseline of the neurologic deficit presented in both patients, which would be useful, in the assessment of the future results of the regimen followed with both patients. Moreover, the degree of their neurologic deficit was quantified using the National Institutes of Health Stroke Scale, a 42-point scale, which tackles 6 major areas of neurologic examination which are: Level of consciousness, visual function, motor function, sensation and neglect, cerebellar function, and language. Both patients were having moderate stroke severity according to the NIHSS scale.

An emergent brain imaging, in the form of a non-contrast CT scan, was done on them after they presented to the ER with acute neurological manifestation, in order to exclude essential mimics such as subarachnoid haemorrhage, intracerebral haemorrhage and masses and to confirm the diagnosis of ischemic stroke.

Although that, The European Heart Rhythm Association guideline recommends the following algorithm for the restart of anticoagulation: 1 day after the transient ischemic attack, 3 days after a mild stroke, 6 days after moderate stroke, and 12 days after a severe stroke, we've given our patients, full dose anticoagulant (enoxaparin 6000 IU SC bid and continue for 5 days and added warfarin (started with 3 mg PO od) guided by INR) from the very first day, in order to demonstrate the benefits of the early initiation of full-dosage anti-coagulant and its positive impact on the morbidity of our patients [18].

A significant improvement was witnessed in both patients either clinically: the first one could walk without support with an improvement of the gaze and sensation and upper limb motor power, after suffering from left hemi-hypoesthesia and lower limb ischemia confirmed by CT angiography on administration. As for the second one, a clinical improvement of the left hemi-hypoesthesia was noticed, confirmed by an improvement on the follow-up CT brain, demonstrating, a nearly complete resolution of the stroke with improvement of MRS.

In a study, published in September 2009, a retrospective trial was conducted to see the benefits & down-comings, of the early bridging, of full dosage anti-coagulant, with patients suffering from an ischemic stroke of moderate intensity, and they have found the following:

Although 10% of enoxaparin, bridging group, experienced parenchymal hematoma within 9–12 days of treatment initiation, a favourable outcome, was experienced, with 69% of the cases, with a mortality rate, of only 1% when compared with a favourable outcome of only 50%, and a mortality rate of 3% of patient who did not receive any anticoagulant treatment. This was contributed to its effect in preventing both stroke progression and recurrence [19].

Another study, published on September 18, consolidated, the continuation of the usage of full dosage anti-coagulant, with patients, coming to ER with a stroke of moderate intensity, as thrombotic events, and recurrence of AIS, have been observed within 90 days in a patient with whom they discontinued, the full dosage anti-coagulant treatment. Adding to this, no increased risk of bleeding was noticed with the continuation of anticoagulant therapy.

Moreover, they shed light, on the strong association between the degree of severity of the stroke and clinical outcomes of the early initiation of full dosage anti-coagulant, as the degree of severity is a strong predictor of success, of the usage of the above-mentioned regimen [20].

Despite the fact that a study, conducted in April 2012, advised against, the early usage of full dosage anti-coagulant, with patients suffering, from ischemic stroke, due to a significant increase in both intracranial and extracranial haemorrhage, the American College of Chest Physicians, advised the usage of this regimen with some sub-types of ischemic strokes: especially those with the cardio-embolic origin, as they found out that the risk of early recurrent strokes is much lower, and that a favourable outcome was observed within 90 days [21].

## 5 Conclusion

In these two cases, the result was positive due to the early start of anticoagulation, but the follow-up time after hospital discharge is not specified either. It is a good start but larger series of patients with a larger study structure are needed.

## Provenance and peer review

Not commissioned, externally peer reviewed.

## Please state any conflicts of interest

None.

## Please state any sources of funding for your research

None.

## Ethical approval

No ethical approval is needed for this type of publication in our institution. However, the patient has given consent to this publication.

## Consent

Written informed consent was obtained from the patient for publication of this case report and accompanying images. A copy of the written consent is available for review by the Editor-in-Chief of this journal on request.

## Author contribution

Mostafa Meshref: study concept or design, surgeon, data collection, data analysis or interpretation, writing the paper, Mostafa Meshref &Nour Shaheen: data collection, data analysis or interpretation, writing the paper, other co-authors: Review and editing.

## Registration of research studies

1. Name of the registry: NA
2. Unique Identifying number or registration ID: NA
3. Hyperlink to your specific registration (must be publicly accessible and will be checked): NA

## Guarantor

Mostafa Meshref.
